# National Influenza Annual Report, Canada, 2022–2023: Canada’s first fall epidemic since the 2019–2020 season

**DOI:** 10.14745/ccdr.v49i10a02

**Published:** 2023-10-01

**Authors:** Kara Schmidt, Myriam Ben Moussa, Steven Buckrell, Abbas Rahal, Taeyo Chestley, Nathalie Bastien, Liza Lee

**Affiliations:** 1 Centre for Immunization and Respiratory Infectious Diseases, Public Health Agency of Canada, Ottawa, ON; 2 National Microbiology Laboratory, Public Health Agency of Canada, Winnipeg, MB

**Keywords:** influenza, epidemic, surveillance, paediatric, influenza A(H3N2), influenza A(H1N1), influenza B, Canada

## Abstract

Coinciding with the beginning of the coronavirus disease 2019 (COVID-19) pandemic in March 2020, Canadian seasonal influenza circulation was suppressed, which was a trend reported globally. Canada saw a brief and delayed return of community influenza circulation during the spring of the 2021–2022 influenza season. Surveillance for Canada’s 2022–2023 seasonal influenza epidemic began in epidemiological week 35 (week starting August 28, 2022) and ended in epidemiological week 34 (week ending August 26, 2023). The 2022–2023 season marked the return to pre-pandemic-like influenza circulation. The epidemic began in epidemiological week 43 (week ending October 29, 2022) and lasted 10 weeks. Driven by influenza A(H3N2), the epidemic was relatively early, extraordinary in intensity, and short in length. This season, a total of 74,344 laboratory-confirmed influenza detections were reported out of 1,188,962 total laboratory tests. A total of 93% of detections were influenza A (n=68,923). Influenza A(H3N2) accounted for 89% of the subtyped specimens (n=17,638/19,876). Late-season, Canada saw community circulation of influenza B for the first time since the 2019–2020 season. The 2022–2023 influenza season in Canada had an extraordinary impact on children and youth; nearly half (n=6,194/13,729, 45%) of reported influenza A(H3N2) detections were in the paediatric (younger than 19 years) population. Weekly paediatric influenza-associated hospital admissions were persistently above historical peak levels for several weeks. The total number of influenza-associated paediatric hospitalizations (n=1,792) far exceeded historical averages (n=1,091). With the return of seasonal influenza circulation and endemic co-circulation of multiple high burden respiratory viruses, sustained vigilance is warranted. Annual seasonal influenza vaccination is a key public health intervention available to protect Canadians.

## Introduction

Globally, comprehensive nonpharmaceutical interventions (NPIs) implemented in March 2020 aimed at reducing the spread of severe acute respiratory syndrome coronavirus 2 (SARS-CoV-2), the virus that causes coronavirus disease 2019 (COVID-19), suppressed seasonal influenza epidemic activity into the period of the usual 2021–2022 Northern Hemisphere season ((1–8)). Canada saw the return of community influenza circulation in the spring of 2022, coinciding with easing of NPIs, which was characterized by a late, low-intensity, and brief seasonal influenza epidemic ((9)). This 2022–2023 influenza season saw the first re-emergence of pre-pandemic-like influenza circulation patterns in Canada ((10)).

Suppressed seasonal influenza activity in recent years, and resulting growing population susceptibility, has raised concerns about the timing, impact, and severity of re-emerging post-pandemic seasonal influenza epidemics ((3,9,11)). Ongoing and timely surveillance plays a critical role in the Public Health Agency of Canada’s ability to respond to influenza and other respiratory virus trends, monitor changes in circulation patterns, and effectively prepare and plan for mitigation measures within the influenza season.

This surveillance report summarizes trends observed during the 2022–2023 influenza season in Canada through analysis of FluWatch core indicators reported by the Public Health Agency of Canada from August 28, 2022 (epidemiological week 35) to August 26, 2023 (epidemiological week 34).

## Methods

FluWatch is Canada’s long-standing influenza surveillance system, which monitors the spread of influenza and influenza-like illness (ILI) through core surveillance indicators based on global epidemiological standards ((12)). FluWatch is a composite surveillance system that consists of eight key areas: virological surveillance; geographic spread; syndromic surveillance; severe outcome surveillance; outbreak surveillance; influenza strain characterization; vaccination coverage; and vaccine effectiveness ((13)). Annually, influenza surveillance is conducted across Canada from epidemiological week 35 to week 34 of the following year. For the 2022–2023 Canadian influenza season, this surveillance period began on August 28, 2022, and ended on August 26, 2023. Detailed methods, including surveillance indicator definitions, data sources and statistical analyses, can be found on the Public Health Agency of Canada’s FluWatch website ((13)).

## Results

### Virological

**Figure 1:**
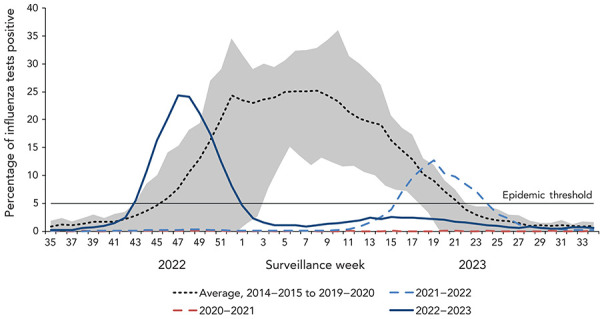
Percentage of influenza tests positive in Canada compared to previous seasons by surveillance week^a^ ^a^ The shaded area represents the maximum and minimum percentage of tests positive reported by week from seasons 2014–2015 to week 11 of 2019–2020. The epidemic threshold is 5% tests positive for influenza. When it is exceeded, and a minimum of 15 weekly influenza detections are reported, a seasonal influenza epidemic is declared

The 2022–2023 national influenza epidemic began early in the season, exceeding the seasonal epidemic threshold (5% or more positive tests and 15 or more detections) in week 43 (late-October). For the second consecutive season, the Canadian influenza epidemic was brief in duration, lasting only 10 weeks, ending week 1 (early-January; **Figure 1**). Compared to pre-pandemic seasons, this tied the earliest start of an epidemic with the 2018–2019 season. The end of the season was unprecedentedly early, as pre-pandemic epidemics consistently ended around week 22 (late-May).

During the 2022–2023 Canadian influenza epidemic, influenza activity peaked in week 47 (late-November) at 24.3% tests positive. This was the first time since the declaration of the COVID-19 pandemic that peak activity approached peak levels observed in pre-pandemic seasons (average 31.3%).

During the 2022–2023 influenza season, a total of 74,344 laboratory-confirmed influenza detections were reported out of 1,188,962 total laboratory tests (**Table 1**). This is both the most detections and most tests ever recorded in a single season, as test counts have increased dramatically from pre-pandemic seasons (average of 276,592 tests and 47,018 detections from seasons 2014–2015 to 2018–2019).

**Table 1:** Number of laboratory tests, detections, and percentage positivity by influenza season, seasons 2014–2015 to 2022–2023, Canada

Season	Influenza tests	Influenza detections	Cumulative percentage of tests positive	Influenza A detections	Influenza B detections	Total influenza A subtyped	Influenza A(H1N1) detections	Influenza A(H3N2) detections
2014–2015	246,930	42,976	17.4%	34,460 (80%)	8,516 (20%)	13,168	94 (1%)	13,074 (99%)
2015–2016	237,826	39,373	16.6%	28,422 (72%)	10,951 (28%)	12,345	11,168 (90%)	1,177 (10%)
2016–2017	267,827	39,365	14.7%	34,848 (89%)	4,517 (11%)	17,747	179 (1%)	17,568 (99%)
2017–2018	319,916	64,337	20.1%	36,103 (56%)	28,234 (44%)	12,443	1,280 (10%)	11,163 (90%)
2018–2019	310,462	49,037	15.8%	46,497 (95%)	2,540 (5%)	17,374	11,606 (67%)	5,768 (33%)
2019–2020	526,483	55,780	10.6%	32,891 (59%)	22,889 (41%)	7,246	4,985 (69%)	2,261 (31%)
2020–2021	666,576	71	0.0%	48 (68%)	23 (32%)	19	6 (32%)	13 (68%)
2021–2022	751,900	16,126	2.1%	15,894 (99%)	232 (1%)	4,734	83 (2%)	4,651 (98%)
2022–2023	1,188,962	74,344	6.3%	68,923 (93%)	5,421 (7%)	19,876	2,238 (11%)	17,638 (89%)

**Figure 2:**
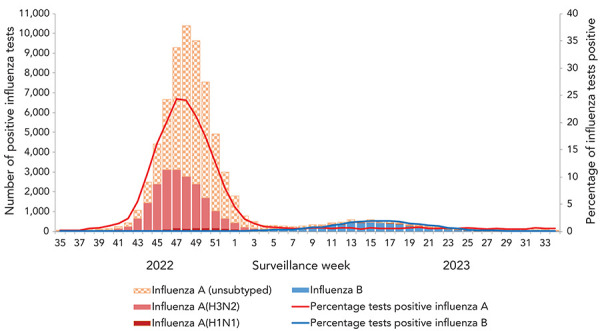
Number of positive influenza tests and percentage of tests positive in Canada, by type, subtype and surveillance week, 2022–2023 influenza season

Influenza A circulated predominantly during the first half of the season and influenza B circulated predominantly in the latter half of the season (**Figure 2**). Overall, a total of 93% of detections were influenza A (n=68,923). Among influenza A subtypes, influenza A(H3N2) predominated, accounting for 89% (n=17,638) of the 19,876 subtyped specimens.

**Figure 3:**
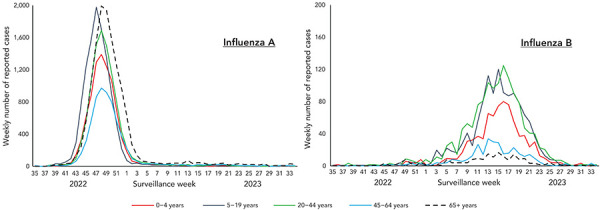
Count of influenza A (left) and influenza B (right) cases in Canada by surveillance week and age group, 2022–2023 influenza season^a^ ^a^ The y-axis scale differs across panels

Detailed information on age and influenza type/subtype was received for 54,096 laboratory-confirmed influenza detections. Influenza A detections were most common among individuals aged 65 years and older (27%; n=13,433), followed by individuals aged 5–19 years (22%; n=11,215). During the 2022–2023 epidemic, the increase in cases in this younger age group preceded increases in all other age groups (**Figure 3**).

Similar to last season, the age distribution of influenza A(H3N2) cases was much younger than pre-pandemic seasons. Nearly half of influenza A(H3N2) detections (45%) were among individuals aged 0–19 years compared to an average of 17% in pre-pandemic seasons (**Table 2**).

**Table 2:** Number and percentage of seasonal influenza A(H3N2) detections by influenza season, by age group, seasons 2014–2015 to 2022–2023, Canada

Age group (years)	Influenza season^a^															
	2014–2015		2015–2016		2016–2017		2017–2018		2018–2019		2019–2020^b^		2021–2022		2022–2023	
	n	%	n	%	n	%	n	%	n	%	n	%	n	%	n	%
0–4	813	6%	79	7%	835	7%	682	7%	275	5%	214	10%	574	19%	2,730	20%
5–19	970	8%	104	10%	1,080	10%	710	7%	506	10%	264	12%	805	27%	3,464	25%
20–44	1,697	14%	175	17%	1,810	16%	1,388	14%	660	13%	344	16%	805	27%	2,647	19%
45–64	1,687	13%	214	20%	1,983	18%	1,595	16%	724	14%	316	15%	293	10%	1,556	11%
65+	7,365	59%	485	46%	5,462	49%	5,882	57%	2,957	58%	981	46%	514	17%	3,332	24%
Total	12,532	N/A	1,057	N/A	11,170	N/A	10,257	N/A	5,122	N/A	2,148	N/A	2,991	N/A	13,729	N/A

Abbreviation: N/A, not applicable

^a^ The 2020–2021 season was excluded from Table 2 as <5 total influenza A(H3N2) cases with age information were reported

^b^ During the 2019–2020 season, case data from one jurisdiction used 20–64-year age group instead of 20–44 and 45–64. These cases have been omitted from the age group-specific case counts but are included in the total case counts

Conversely, influenza B detections were least common among individuals aged 65 years and older (5%; n=196) and 45–64 years (8%; n=327; **Table 3**). A similar case age distribution was observed in pre-pandemic seasons where influenza B Victoria lineage predominated over Yamagata lineage. In each of these seasons, cases occurred least frequently in these older age groups.

**Table 3:** Number and percentage of seasonal influenza B detections by influenza season, by age group, seasons 2014–2015 to 2022–2023, Canada

Age group (years)	Influenza season (predominant influenza B lineage)^a,b^															
	2014–2015 Yamagata		2015–2016 Victoria		2016–2017 Yamagata		2017–2018 Yamagata		2018–2019 Victoria		2019–2020 Victoria^c^		2021–2022 N/A^d^		2022–2023 Victoria	
	n	%	n	%	n	%	n	%	n	%	n	%	n	%	n	%
0–4	569	8%	1,800	19%	293	9%	1,615	7%	379	20%	4,170	22%	43	22%	807	21%
5–19	810	11%	2,765	29%	549	17%	2,994	13%	638	34%	6,094	32%	28	14%	1,233	31%
20–44	1,157	16%	2,262	24%	536	17%	3,051	13%	434	23%	5,737	30%	46	24%	1,361	35%
45–64	1,850	25%	1,150	12%	737	23%	5,098	21%	144	8%	1,203	6%	27	14%	327	8%
65+	2,935	40%	1,640	17%	1,053	33%	11,015	46%	276	15%	1,455	8%	51	26%	196	5%
Total	7,321	N/A	9,617	N/A	3,168	N/A	23,773	N/A	1,871	N/A	18,878	N/A	195	N/A	3,924	N/A

Abbreviation: N/A, not applicable

^a^ The 2020–2021 season was excluded from Table 3 as <5 total influenza B cases with age information were reported

^b^ Predominant lineage was determined by influenza B specimen antigenic characterization performed by the National Microbiology Laboratory. In each season, >75% of characterized specimens belonged to either Victoria or Yamagata lineage with predominance attributed to the lineage exceeding this threshold

^c^ During the 2019–2020 season, case data from one jurisdiction used 20–64 years age group instead of 20–44 and 45–64. These cases have been omitted from the age group-specific case counts but are included in the total case counts

^d^ There were no influenza B specimens received and characterized by the National Microbiology Laboratory during the 2021–2022 season, therefore no predominant lineage could be assigned

## Influenza/influenza-like illness activity levels

**Figure 4:**
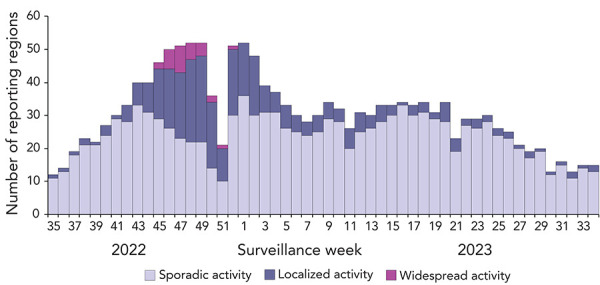
Number of influenza surveillance regions reporting sporadic, localized, or widespread activity by surveillance week in Canada, 2022–2023 influenza season

Sporadic influenza activity was reported by at least 10 reporting regions in each week of the 2022–2023 season. Localized activity was also reported by at least one reporting region in each week of the 2022–2023 influenza season. Coinciding with peak percent positivity observed in FluWatch’s virological data, national influenza activity levels peaked between weeks 45 and 52 (early-November to late-December), where widespread activity was reported every week (**Figure 4**). No widespread influenza activity was reported after week 52 (late-December). The sharp decline at weeks 50 and 51 coincided with the holiday season, where data was not reported by many regions.

## Syndromic-sentinel primary healthcare provider influenza-like illness surveillance

During the 2022–2023 influenza season, a weekly average of 3,144 patients were seen by a weekly average of 42 sentinel primary care providers. Both metrics were lower than the 2021–2022 season, where an average of 50 sentinel primary care providers saw a weekly average of 3,769 patients.

**Figure 5:**
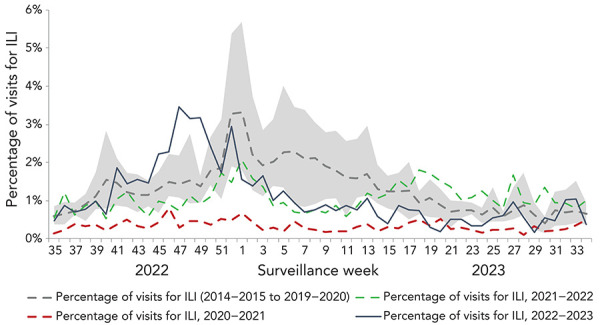
Percentage of visits for influenza-like illness reported by sentinel primary care providers in Canada by season and surveillance week^a^ Abbreviation: ILI, influenza-like illness ^a^ The shaded area represents the maximum and minimum percentage of visits for ILI reported by week from seasons 2014–2015 to week 11 of 2019–2020

During that season, the weekly percentage of visits to primary care providers for ILI followed expected trends, ranging between 0.2% and 3.5% (**Figure 5**). The percentage of weekly visits for ILI remained within historical levels until week 45 (early-November), peaked in week 47 (late-November) at 3.5%, and remained above historical levels until week 51 (late-December). Influenza-like illness visits remained within or below historical levels for the remainder of the 2022–2023 season. Influenza-like illness visit trends coincided with increases in influenza activity and ultimately reflected the timing of the 2022–2023 influenza season.

## Syndromic-FluWatchers

**Figure 6:**
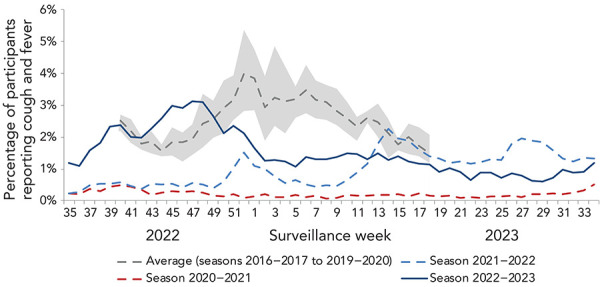
Percentage of FluWatcher participants reporting cough and fever in Canada by season and surveillance week^a^ ^a^ The shaded area represents the maximum and minimum percentage of FluWatcher participants reporting influenza-like illness by week from seasons 2016–2017 to week 11 of 2019–2020

During the 2022–2023 season, an average of 10,142 FluWatchers reported each week, with a total of 15,755 FluWatchers participating over the season and a total of 527,363 questionnaires submitted. The percentage of FluWatchers reporting ILI symptoms (acute onset of cough and fever) surpassed historical levels in week 42 (mid-October), peaked in week 47 (late-November) at 3.1%, and remained above historical levels until week 48 (early-December; **Figure 6**). Levels gradually decreased and remained below expected levels until the end of the 2022–2023 season. Self-reported ILI did not increase significantly over the period of influenza B circulation.

**Figure 7:**
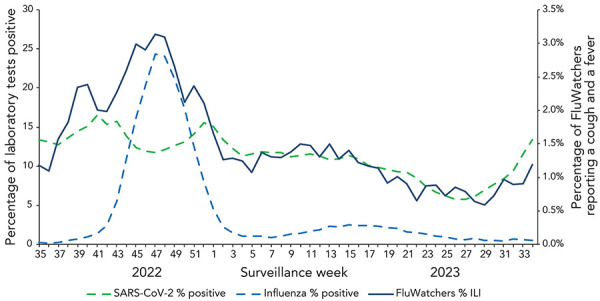
Percentage of influenza and SARS-CoV-2 laboratory tests positive and percentage of FluWatchers reporting cough and fever in Canada by surveillance week, 2022–2023 influenza season Abbreviations: ILI, influenza-like illness; SARS-CoV-2, severe acute respiratory syndrome coronavirus 2

The reports of ILI are not specific to any one respiratory pathogen and can be due to influenza or other respiratory viruses, including SARS-CoV-2. This makes the proportion of FluWatchers reporting ILI an important indicator of overall respiratory illness activity in the community. The percentage of FluWatchers reporting ILI captured trends in laboratory-confirmed respiratory virus detections, notably of SARS-CoV-2 and influenza. Increases in self-reported ILI tend to mirror increases in both SARS-CoV-2 percent positivity as well as influenza percent positivity (**Figure 7**).

## Outbreaks

During the 2022–2023 season, 626 laboratory-confirmed influenza outbreaks were reported, with the majority occurring in long-term care facilities (LTCFs) (53.5%), followed by facilities categorized as ”other“ (28.6%); **Table 4**). The number and proportion (n=335, 53.5%) of laboratory-confirmed influenza outbreaks occurring in LCTFs was lower than recent pre-pandemic seasons (n=639, 62% in 2018–2019; n=615, 64% in 2019–2020). This may be related to differences in reporting among provinces and territories compared to previous seasons. The number of laboratory-confirmed outbreaks reported in a week peaked in week 49 (early-December; n=84), which coincided with the peak of the influenza season.

**Table 4:** Number and percentage of laboratory-confirmed influenza outbreaks in Canada by setting and season, seasons 2018–2019 to 2020–2023

Year	Long-term care facilities		Acute care facilities		Schools and daycares		Remote or isolated communities		Other	
	n	%	n	%	n	%	n	%	n	%
2018–2019	639	61.6	138	13.3	32	3.1	N/A	N/A	229	22.1
2019–2020	615	64.2	89	9.3	15	1.6	0	0	239	24.9
2020–2021	0	0	0	0	0	0	0	0	0	0
2021–2022	45	51.1	5	5.7	1	1.1	3	3.4	34	38.6
2022–2023	335	53.5	101	16.1	4	0.6	7	1.1	179	28.6

Abbreviation: N/A, not applicable

## Severe outcomes-provincial/territorial severe outcome surveillance

During the 2022–2023 season, 4,216 influenza-associated hospitalizations were reported by participating provinces and territories. Most hospitalizations were associated with influenza A (97%), and among hospitalizations with subtype information, 85% (n=1,804) were associated with influenza A(H3N2).

**Figure 8:**
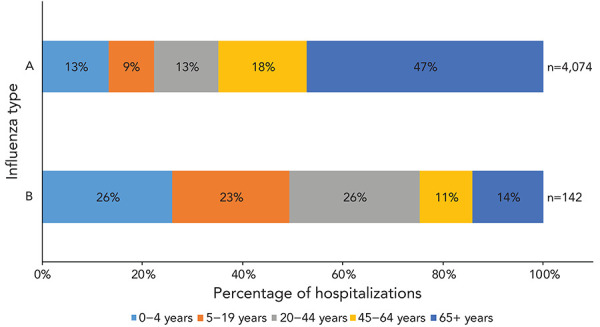
Age distribution of hospitalizations by influenza type in Canada, 2022–2023 influenza season

The annual seasonal hospitalization incidence for the 2022–2023 season was 49 hospitalizations per 100,000 population, which is within values recorded in previous seasons (**Table 5**). Among hospitalizations, heterogeneity existed between age groups. The highest cumulative hospitalization rates were among children aged 0–4 years (131 per 100,000 population) and adults aged 65 years and older (131 per 100,000 population). These rates significantly exceeded both the cumulative rates among remaining age groups, a trend observed in last season’s predominant influenza A(H3N2) epidemic. However, in pre-pandemic seasons of predominant influenza A(H3N2) circulation, hospitalization rates were much higher among adults aged 65 years and older, relative to younger age groups. Influenza A accounted for the vast majority of hospitalizations. When hospitalizations are broken down by type, the paediatric population (19 years and younger) accounted for 49% of hospitalizations associated with influenza B compared to 22% of hospitalizations associated with influenza A (**Figure 8**).

**Table 5:** Estimated annual seasonal incidence of influenza hospitalizations, per 100,000 population, in Canada by season and age group, seasons 2014–2015 to 2022–2023^a^

Age group (years)	Influenza season (predominant influenza A subtype)							
	2014–2015 (H3N2)	2015–2016 (H1N1)	2016–2017 (H3N2)	2017–2018 (H3N2)	2018–2019 (H1N1)	2019–2020 (H1N1)	2021–2022 (H3N2)	2022–2023 (H3N2)
0–4	46	86^b^	46	70	98	77^b^	19	131^b^
5–19	10	14	9	17	21	16	7	27
20–44	6	10	5	12	15	14	5	19
45–64	16	23	15	41	40	23	6	33
65+	175^b^	52	128^b^	280^b^	127^b^	76	21^b^	131^b^
Overall	37	25	30	64	45	30	9	49

^a^ The 2020–2021 season was excluded from Table 5 as no influenza hospitalizations were reported

^b^ The shaded cells highlight the age group with the highest estimated incidence of influenza hospitalizations for the respective season

This season, 362 intensive care unit (ICU) admissions and 275 deaths were reported by participating provinces and territories. Intensive care unit admissions were most common among adults aged 65 years and older (32%) and 45–64 years of age (28%). Deaths were most common among adults aged 65 years and older (76%). The percentage of hospitalizations that resulted in ICU admissions was comparable to values reported in historical seasons (**Table 6**).

**Table 6:** Percentage of hospitalizations that resulted in intensive care unit admissions in Canada by season and age group, seasons 2014–2015 to 2022–2023^a^

Age group (years)	Influenza season							
	2014–2015	2015–2016	2016–2017	2017–2018	2018–2019	2019–2020	2021–2022	2022–2023
0–4	4%	5%	4%	13%	12%	10%	10%	9%
5–19	6%	6%	6%	18%	13%	14%	6%	7%
20–44	9%	14%	8%	14%	22%	10%	11%	11%
45–64	10%	17%	9%	19%	28%	20%	13%	14%
65+	4%	7%	3%	7%	12%	10%	7%	6%
Overall	5%	10%	4%	10%	17%	12%	9%	9%

^a^ The 2020–2021 season was excluded from Table 6 as no influenza hospitalizations were reported

## Severe outcomes—Canadian Immunization Monitoring Program, ACTive

The Canadian Immunization Monitoring Program, ACTive (IMPACT) network preliminarily reported 1,792 influenza-associated paediatric hospitalizations during the 2022–2023 influenza season, which was greater than historical seasons. From 2014–2015 to 2019–2020, an average of 1,091 paediatric hospitalizations were reported, with 1,354 hospitalizations being the highest reported in a single season (2018–2019).

**Figure 9:**
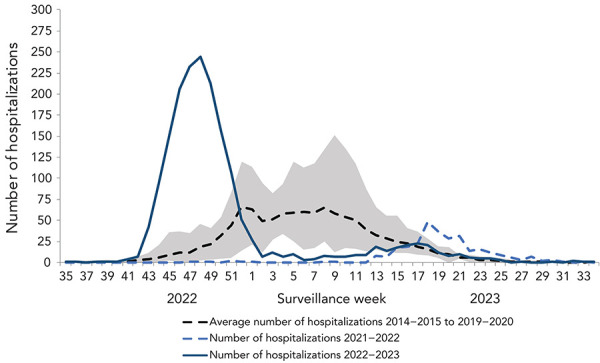
Preliminary weekly number of paediatric hospitalizations in Canada, reported by IMPACT by season and week of admission^a^ Abbreviation: IMPACT, Canadian Immunization Monitoring Program, ACTive ^a^ The shaded area represents the maximum and minimum number of paediatric hospitalizations reported by IMPACT, by week from seasons 2014–2015 to week 11 of 2019–2020

Weekly preliminary paediatric hospitalizations rapidly increased as of week 42 (mid-October) before reaching a peak in week 48 (early-December; n=242; **Figure 9**). This peak was early and of extraordinary intensity. Pre-pandemic (seasons 2014–2015 to 2019–2020), paediatric hospitalizations peaked no earlier than at week 52, at an average of 66 hospitalizations.

**Figure 10:**
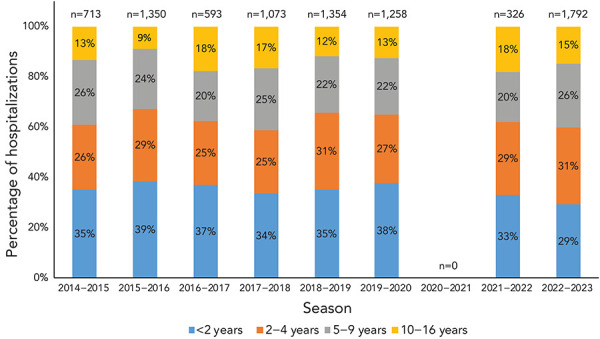
Age distribution of paediatric hospitalizations in Canada reported by IMPACT, seasons 2014–2015 to 2022–2023 Abbreviation: IMPACT, Canadian Immunization Monitoring Program, ACTive

Most hospitalizations (n=1,612, 90%) were associated with influenza A. Among hospitalizations for which influenza subtype was available, 94% (n=643) were associated with influenza A(H3N2). The overall age distribution of paediatric hospitalizations was not vastly different compared to previous seasons (**Figure 10**). However, for the first time over the last seven influenza epidemics, the proportion of hospitalized cases aged 2–4 years was highest, rather than their younger cohort younger than 2 years of age. The total number and the age distribution of paediatric influenza B-associated hospitalizations were within ranges seen in pre-pandemic seasons (**Table 7**).

**Table 7:** Number and percentage of paediatric influenza B-associated hospitalizations in Canada reported by IMPACT by age group, seasons 2014–2015 to 2022–2023^a^

Age group (years)	Influenza season													
	2014–2015		2015–2016		2016–2017		2017–2018		2018–2019		2019–2020		2022–2023	
	n	%	n	%	n	%	n	%	n	%	n	%	n	%
<2	60	30%	138	30%	31	24%	101	25%	32	25%	199	33%	50	28%
2–4	53	26%	118	26%	32	25%	92	23%	44	35%	161	26%	51	28%
5–9	54	27%	145	31%	39	30%	127	31%	34	27%	162	27%	58	32%
10–16	35	17%	60	13%	28	22%	84	21%	17	13%	89	15%	21	12%
Total	202	100%	461	100%	130	100%	404	100%	127	100%	611	100%	180	100%

Abbreviation: IMPACT, Canadian Immunization Monitoring Program, ACTive

^a^ The 2020–2021 and 2021–2022 seasons were excluded from Table 7 comparison as no influenza hospitalizations and <5 influenza hospitalizations were reported, respectively

This season, 283 ICU admissions and 10 deaths were reported. The highest proportion of ICU admissions was reported among cases aged 2–4 years (29%) and 10–16 years (22%). The percentage of paediatric hospitalizations that resulted in ICU admissions was comparable to values reported in historical seasons (**Table 8**).

**Table 8:** Percentage of paediatric hospitalizations that resulted in intensive care unit admissions in Canada reported by IMPACT by age group, seasons 2014–2015 to 2022–2023^a^

Age group (years)	Influenza season							
	2014–2015	2015–2016	2016–2017	2017–2018	2018–2019	2019–2020	2021–2022	2022–2023
<2	10%	13%	13%	17%	17%	16%	8%	16%
2–4	20%	17%	12%	16%	20%	19%	10%	15%
5–9	14%	19%	19%	20%	24%	18%	9%	12%
10–16	22%	29%	29%	26%	24%	25%	19%	23%
Overall	15%	17%	17%	19%	20%	18%	11%	16%

Abbreviation: IMPACT, Canadian Immunization Monitoring Program, ACTive

^a^ The 2020–2021 season was excluded from Table 8 as no influenza hospitalizations were reported

### Influenza strain characterization

From September 1, 2022, to August 31, 2023, the National Microbiology Laboratory characterized 684 influenza viruses (460 A(H3N2), 108 A(H1N1) and 116 influenza B) that were received from Canadian laboratories.

### Genetic characterization influenza A(H3N2)

Ten influenza A(H3N2) viruses did not grow to sufficient hemagglutination titers for antigenic characterization by hemagglutination inhibition (HI) assays. Therefore, the National Microbiology Laboratory performed genetic characterization to determine the genetic group identity of these viruses. Sequence analysis of the hemagglutinin (HA) genes of the viruses showed that they belonged to genetic group 3C.2a1b.2a2. The A/Darwin/6/2021 (H3N2)-like virus is an influenza A(H3N2) component of the 2022–2023 Northern Hemisphere influenza vaccine and belongs to genetic group 3C.2a1b.2a2.

### Antigenic characterization

#### Influenza A(H3N2)

Of the 450 influenza A(H3N2) viruses characterized, 441 were characterized as antigenically similar to A/Darwin/6/2021 (H3N2)-like virus with antisera raised against cell-grown A/Darwin/6/2021 (H3N2)-like virus. Nine viruses showed reduced titer with antisera raised against cell-grown A/Darwin/6/2021 (H3N2)-like virus. The A/Darwin/6/2021 (H3N2)-like virus is an influenza A(H3N2) component of the 2022–2023 Northern Hemisphere influenza vaccine. The 450 influenza A(H3N2) viruses characterized belonged to genetic group 3C.2a1b.2a2.

#### Influenza A(H1N1)

The 108 influenza A(H1N1) viruses were characterized as antigenically similar to A/Wisconsin/588/2019-like with ferret antisera produced against cell-propagated A/Wisconsin/588/2019. The A/Wisconsin/588/2019 is the influenza A(H1N1) component of the 2022–2023 Northern Hemisphere influenza vaccine.

#### Influenza B

Influenza B viruses can be divided into two antigenically distinct lineages represented by B/Yamagata/16/88 and B/Victoria/2/87 viruses. The recommended influenza B components for the 2022–2023 Northern Hemisphere influenza vaccine are B/Austria/1359417/2021 (Victoria lineage) and B/Phuket/3073/2013 (Yamagata lineage). The 116 viruses characterized were antigenically similar to B/Austria/1359417/2021.

### Antiviral resistance

The 604 influenza viruses (383 A(H3N2), 106 A(H1N1) and 115 influenza B) were tested for antiviral resistance, with 100% of viruses sensitive to oseltamivir and zanamivir.

## Vaccination coverage

Influenza vaccination coverage among all adults for the 2022–2023 influenza season (43%) was slightly higher than the previous season (39%). Among those at higher risk of complications from influenza (adults aged 65 years and older and adults aged 18–64 years with chronic medical conditions), vaccination coverage was 74% and 43% respectively, both similar to the previous season and below Canada’s influenza vaccination coverage goal of 80% for those at higher risk ((14)).

## Vaccine effectiveness

The Canadian Sentinel Practitioner Surveillance Network provides estimates of the effectiveness of the seasonal influenza vaccine in preventing medically attended illness due to laboratory-confirmed influenza among Canadians ((15)). Based on data collected between November 1, 2022, and January 6, 2023, vaccine effectiveness was estimated to be 54% against influenza A(H3N2). Due to the dominant circulation of influenza A(H3N2) this season, the vaccine effectiveness estimate was only available for one influenza subtype. By age group, vaccine effectiveness was 47% (95% CI: 11–69) for individuals under the age of 19 years, 58% (95% CI: 33–73) for adults aged 20–64 years and 59% (95% CI: 15–80) for adults aged 65 years and older.

## Discussion

The 2022–2023 influenza epidemic in Canada, driven by influenza A(H3N2), was early, intense, and had an extraordinary impact on children and adolescents ((10)). The national influenza epidemic began in week 43 (late-October), peaked rapidly in week 47 (late-November), and ended unprecedentedly early in week 1 (early-January). Early and intense activity with influenza A(H3N2) predominance was also seen in the United States and Europe this season and in regions of the Southern Hemisphere during their 2022 influenza season ((16–19)). The intensity of this season’s influenza epidemic coincided with unusually early respiratory syncytial virus (RSV) activity and ongoing SARS-CoV-2 circulation, which posed a threat to public health and increased pressures on the Canadian healthcare system.

The dominance of influenza A(H3N2) seen during the 2021–2022 Canadian influenza season continued into the 2022–2023 season. Similar to last season, several FluWatch indicators demonstrated that the paediatric population was atypically afflicted. For the second straight season, nearly half of influenza A(H3N2) cases were aged 0–19 years, more than double the average recorded in pre-pandemic years. Additionally, hospitalization rates were once again similar among children aged 0–4 years and adults aged 65 years and older, a distribution not observed during pre-pandemic influenza A(H3N2) predominant epidemics, where burden is typically highest in older adults. Perhaps most notable, the total number of influenza-associated paediatric hospitalizations preliminarily reported by IMPACT during the 2022–2023 influenza season greatly exceeded the total reported in any pre-pandemic season. Weekly paediatric influenza-associated hospitalization admissions were persistently higher than historical peak levels for several weeks during the 2022–2023 season. As was previously hypothesized, the atypical age distribution may reflect immunologic factors ((9,10)). A large, unexposed cohort of young children may have been more vulnerable to infection following the suppression of seasonal respiratory virus transmission across Canada in recent years. The percentage of hospitalizations in both paediatrics and adults that resulted in ICU admissions was within values previously reported, suggesting that despite the high number of hospitalizations this season, they were not necessarily more severe.

As the 2022–2023 influenza epidemic waned, so did the dominance of influenza A(H3N2), as increased detections of influenza A(H1N1) and influenza B were observed, which was a trend also seen in other Northern Hemisphere regions ((17,20)). The small wave of influenza B that occurred later in the season mirrored pre-pandemic patterns with its timing. The National Microbiology Laboratory characterized and classified all influenza B viruses as belonging to B/Victoria lineage. As of February 2023, it was reported by the World Health Organization that there had been no confirmed detections of circulating B/Yamagata lineage viruses since before April 2020 ((21)). Historically, in Canada, the age distribution of influenza B cases has differed between influenza B/Victoria and influenza B/Yamagata predominant seasons. In pre-pandemic seasons, where influenza B/Victoria predominated, the majority of influenza B cases were younger than 45 years of age, while the opposite was true of influenza B/Yamagata predominant seasons. This trend has been reported elsewhere and was notable through the 2022–2023 influenza season in Canada, with 87% of influenza B cases younger than 45 years of age ((22–25)). If influenza B/Yamagata community circulation does not return, there may be future implications for how populations are affected by influenza B.

Canada has not observed widespread circulation of influenza A(H1N1) since the 2019–2020 season, leaving a large unexposed cohort of the general population, especially new cohorts of children younger than four years. The 2023 summer saw waning dominance of influenza A(H3N2) globally, and a resurgence of influenza A(H1N1) activity in the upcoming season is possible. However, an abundance of factors can influence influenza activity and severity: antigenic drift, co-circulation of other respiratory viruses, vaccination coverage, vaccine effectiveness, antiviral use, population imprinting, cohort effects, and contextual factors ((25–33)).

Though the younger cohort was unusually impacted during the past two influenza epidemics in Canada ((9,10)), adults with chronic health conditions and older adults remain at high risk of severe outcomes. With endemic co-circulation of multiple high burden respiratory viruses impacting all age groups (influenza, SARS-CoV-2, RSV), and potential emergence of non-seasonal respiratory viruses, the importance of respiratory virus surveillance in Canada is highlighted. Predicting influenza activity is notoriously difficult, and this can be mitigated with comprehensive surveillance activities and the use of historical data and trends to determine likely outcomes to in-season observations. Sustained vigilance and integrated planning approaches for upcoming predictably unpredictable respiratory virus seasons, in the context of a strained healthcare system, are essential ((3,29)).

Influenza can cause severe illness across all age groups, with or without chronic health conditions ((25)). Certain populations, such as young children, older adults, individuals with chronic health conditions, residents of LTCF and chronic care facilities, pregnant individuals, and Indigenous peoples are at greater risk of serious complications or worsening of underlying health conditions ((34)). Annual influenza vaccination remains a critical tool for the prevention of influenza and its complications, and reduced transmissibility to others.

## References

[1] Lee L, Butt K, Buckrell S, Nwosu A, Sevenhuysen C, Bancej C. National influenza mid-season report, 2020-2021. Can Commun Dis Rep 2021;47(1):1–4. 10.14745/ccdr.v47i01a01 33679244 PMC7919779

[2] Nwosu A, Lee L, Schmidt K, Buckrell S, Sevenhuysen C, Bancej C. National influenza annual report, Canada, 2020-2021, in the global context. Can Commun Dis Rep 2021;47(10):405–13. 10.14745/ccdr.v47i10a02 34737672 PMC8525606

[3] Bancej C, Rahal A, Lee L, Buckrell S, Schmidt K, Bastien N. National FluWatch mid-season report, 2021-2022: sporadic influenza activity returns. Can Commun Dis Rep 2022;48(1):39–45. 10.14745/ccdr.v48i01a06 35273468 PMC8856831

[4] Tang JW, Bialasiewicz S, Dwyer DE, Dilcher M, Tellier R, Taylor J, Hua H, Jennings L, Kok J, Levy A, Smith D, Barr IG, Sullivan SG. Where have all the viruses gone? Disappearance of seasonal respiratory viruses during the COVID-19 pandemic. J Med Virol 2021;93(7):4099–101. 10.1002/jmv.26964 33760278 PMC8250511

[5] Sullivan SG, Carlson S, Cheng AC, Chilver MB, Dwyer DE, Irwin M, Kok J, Macartney K, MacLachlan J, Minney-Smith C, Smith D, Stocks N, Taylor J, Barr IG. Where has all the influenza gone? The impact of COVID-19 on the circulation of influenza and other respiratory viruses, Australia, March to September 2020. Euro Surveill 2020;25(47):2001847. 10.2807/1560-7917.ES.2020.25.47.2001847 33243355 PMC7693168

[6] Olsen SJ, Azziz-Baumgartner E, Budd AP, Brammer L, Sullivan S, Pineda RF, Cohen C, Fry AM. Decreased influenza activity during the COVID-19 pandemic-United States, Australia, Chile, and South Africa, 2020. Am J Transplant 2020;20(12):3681–5. 10.1111/ajt.16381 33264506 PMC7753605

[7] Groves HE, Papenburg J, Mehta K, Bettinger JA, Sadarangani M, Halperin SA, Morris SK; for members of the Canadian Immunization Monitoring Program Active (IMPACT). The effect of the COVID-19 pandemic on influenza-related hospitalization, intensive care admission and mortality in children in Canada: A population-based study. Lancet Reg Health Am 2022;7:100132. 10.1016/j.lana.2021.100132 35291567 PMC8913102

[8] Groves HE, Piché-Renaud PP, Peci A, Farrar DS, Buckrell S, Bancej C, Sevenhuysen C, Campigotto A, Gubbay JB, Morris SK. The impact of the COVID-19 pandemic on influenza, respiratory syncytial virus, and other seasonal respiratory virus circulation in Canada: A population-based study. Lancet Reg Health Am 2021;1:100015. 10.1016/j.lana.2021.100015 34386788 PMC8285668

[9] Buckrell S, Ben Moussa M, Bui T, Rahal A, Schmidt K, Lee L, Bastien N, Bancej C. National Influenza Annual Report, Canada, 2021-2022: A brief, late influenza epidemic. Can Commun Dis Rep 2022;48(10):473–83. 10.14745/ccdr.v48i10a07 38125392 PMC10730107

[10] Ben Moussa M, Buckrell S, Rahal A, Schmidt K, Lee L, Bastien N, Bancej C. National influenza mid-season report, 2022-2023: A rapid and early epidemic onset. Can Commun Dis Rep 2023;49(1):10–4. 10.14745/ccdr.v49i01a03 36815865 PMC9902033

[11] Baker RE, Park SW, Yang W, Vecchi GA, Metcalf CJ, Grenfell BT. The impact of COVID-19 nonpharmaceutical interventions on the future dynamics of endemic infections. Proc Natl Acad Sci USA 2020;117(48):30547–53. 10.1073/pnas.2013182117 33168723 PMC7720203

[12] World Health Organization (WHO). Global epidemiological surveillance standards for influenza. Geneva (CH): WHO; 2013. https://www.who.int/publications/i/item/9789241506601

[13] Public Health Agency of Canada. Overview of influenza monitoring in Canada. Ottawa, ON: PHAC. [Modified 2019 Dec 10]. https://www.canada.ca/en/public-health/services/diseases/flu-influenza/influenza-surveillance/about-fluwatch.html#a2.4

[14] Public Health Agency of Canada. Highlights from the 2022–2023 Seasonal influenza (flu) vaccination coverage survey. Ottawa, ON: PHAC. [Modified 2023 July 6]. https://www.canada.ca/en/public-health/services/immunization-vaccines/vaccination-coverage/seasonal-influenza-survey-results-2022-2023.html

[15] Skowronski DM, Chuang ES, Sabaiduc S, Kaweski SE, Kim S, Dickinson JA, Olsha R, Gubbay JB, Zelyas N, Charest H, Bastien N, Jassem AN, De Serres G. Vaccine effectiveness estimates from an early-season influenza A(H3N2) epidemic, including unique genetic diversity with reassortment, Canada, 2022/23. Euro Surveill 2023;28(5):2300043. 10.2807/1560-7917.ES.2023.28.5.2300043 36729117 PMC9896608

[16] Centers for Disease Control and Prevention (CDC). Early wave of flu brings early flu hospitalizations. Atlanta, GA: CDC. [Modified 2022 Oct 28]. https://www.cdc.gov/flu/spotlights/2022-2023/early-wave-hospitalizations.htm

[17] European Centre for Disease Prevention and Control/WHO Regional Office for Europe. Season overview. Flu News Europe. [Accessed 2023 Aug 16]. https://flunewseurope.org/SeasonOverview

[18] Olivares Barraza MF, Fasce RA, Nogareda F, Marcenac P, Vergara Mallegas N, Bustos Alister P, Loayza S, Chard AN, Arriola CS, Couto P, García Calavaro C, Rodriguez A, Wentworth DE, Cuadrado C, Azziz-Baumgartner E. Influenza incidence and vaccine effectiveness during the southern hemisphere influenza season – Chile, 2022. MMWR Morb Mortal Wkly Rep 2022;71(43):1353–8. 10.15585/mmwr.mm7143a1 36301733 PMC9620570

[19] Australian Government. AISR – 2022 national influenza season summary. Australian Government Department of Health and Aged Care; 2022. https://www.health.gov.au/resources/publications/aisr-2022-national-influenza-season-summary?language=en

[20] Centers for Disease Control and Prevention (CDC). Weekly U.S. influenza surveillance report. Atlanta, GA: CDC. [Accessed 2023 Aug 1]. https://www.cdc.gov/flu/weekly/index.htm

[21] World Health Organization (WHO). Recommended composition of influenza virus vaccines for use in the 2023-2024 northern hemisphere influenza season. WHO; 2023. https://cdn.who.int/media/docs/default-source/influenza/who-influenza-recommendations/vcm-northern-hemisphere-recommendation-2023-2024/202302_seasonal_recommendation_a.pdf?sfvrsn=42612ae5_3&download=true

[22] Jung SW, Kim YJ, Han SB, Lee KY, Kang JH. Differences in the age distribution of influenza B virus infection according to influenza B virus lineages in the Korean population. Postgrad Med 2021;133(1):82–8. 10.1080/00325481.2020.1825295 32945235

[23] Yang J, Lau YC, Wu P, Feng L, Wang X, Chen T, Ali ST, Peng Z, Fang VJ, Zhang J, He Y, Lau EH, Qin Y, Yang J, Zheng J, Jiang H, Yu H, Cowling BJ. Variation in influenza B virus epidemiology by lineage, China. Emerg Infect Dis 2018;24(8):1536–40. 10.3201/eid2408.180063 30015611 PMC6056115

[24] Caini S, Kusznierz G, Garate VV, Wangchuk S, Thapa B, de Paula Júnior FJ, Ferreira de Almeida WA, Njouom R, Fasce RA, Bustos P, Feng L, Peng Z, Araya JL, Bruno A, de Mora D, Barahona de Gámez MJ, Pebody R, Zambon M, Higueros R, Rivera R, Kosasih H, Castrucci MR, Bella A, Kadjo HA, Daouda C, Makusheva A, Bessonova O, Chaves SS, Emukule GO, Heraud JM, Razanajatovo NH, Barakat A, El Falaki F, Meijer A, Donker GA, Huang QS, Wood T, Balmaseda A, Palekar R, Arévalo BM, Rodrigues AP, Guiomar R, Lee VJ, Ang LW, Cohen C, Treurnicht F, Mironenko A, Holubka O, Bresee J, Brammer L, Le MT, Hoang PV, El Guerche-Séblain C, Paget J; Global Influenza B Study team. The epidemiological signature of influenza B virus and its B/Victoria and B/Yamagata lineages in the 21st century. PLoS One 2019;14(9):e0222381. 10.1371/journal.pone.0222381 31513690 PMC6742362

[25] Andrew MK, Pott H, Staadegaard L, Paget J, Chaves SS, Ortiz JR, McCauley J, Bresee J, Nunes MC, Baumeister E, Raboni SM, Giamberardino HI, McNeil SA, Gomez D, Zhang T, Vanhems P, Koul PA, Coulibaly D, Otieno NA, Dbaibo G, Almeida ML, Laguna-Torres VA, Drăgănescu AC, Burtseva E, Sominina A, Danilenko D, Medić S, Diez-Domingo J, Lina B. Age differences in comorbidities, presenting symptoms, and outcomes of influenza illness requiring hospitalization: A worldwide perspective from the global influenza hospital surveillance network. Open Forum Infect Dis 2023;10(6):ofad244. 10.1093/ofid/ofad244 37383245 PMC10296081

[26] Skowronski DM, Leir S, De Serres G, Murti M, Dickinson JA, Winter AL, Olsha R, Croxen MA, Drews SJ, Charest H, Martineau C, Sabaiduc S, Bastien N, Li Y, Petric M, Jassem A, Krajden M, Gubbay JB. Children under 10 years of age were more affected by the 2018/19 influenza A(H1N1)pdm09 epidemic in Canada: ‎possible cohort effect following the 2009 influenza pandemic. Euro Surveill 2019;24(15):1900104. 10.2807/1560-7917.ES.2019.24.15.1900104 30994107 PMC6470369

[27] Chen Z, Bancej C, Lee L, Champredon D. Antigenic drift and epidemiological severity of seasonal influenza in Canada. Sci Rep 2022;12(1):15625. 10.1038/s41598-022-19996-7 36115880 PMC9482630

[28] Axelsen JB, Yaari R, Grenfell BT, Stone L. Multiannual forecasting of seasonal influenza dynamics reveals climatic and evolutionary drivers. Proc Natl Acad Sci USA 2014;111(26):9538–42. 10.1073/pnas.1321656111 24979763 PMC4084473

[29] Lagacé-Wiens P, Bullard J, Cole R, Van Caeseele P. Seasonality of coronaviruses and other respiratory viruses in Canada: implications for COVID-19. Can Commun Dis Rep 2021;47(3):132–8. 10.14745/ccdr.v47i03a02 34012336 PMC8109286

[30] Gagnon A, Acosta E, Miller MS. Age-specific incidence of influenza A responds to change in virus subtype dominance. Clin Infect Dis 2020;71(7):e195–8. 10.1093/cid/ciaa075 31985006

[31] Gostic KM, Bridge R, Brady S, Viboud C, Worobey M, Lloyd-Smith JO. Childhood immune imprinting to influenza A shapes birth year-specific risk during seasonal H1N1 and H3N2 epidemics. PLoS Pathog 2019;15(12):e1008109. 10.1371/journal.ppat.1008109 31856206 PMC6922319

[32] Budd AP, Beacham L, Smith CB, Garten RJ, Reed C, Kniss K, Mustaquim D, Ahmad FB, Cummings CN, Garg S, Levine MZ, Fry AM, Brammer L. Birth cohort effects in influenza surveillance data: evidence that first influenza infection affects later influenza-associated illness. J Infect Dis 2019;220(5):820–9. 10.1093/infdis/jiz201 31053844 PMC6669091

[33] Arevalo P, McLean HQ, Belongia EA, Cobey S. Earliest infections predict the age distribution of seasonal influenza A cases. eLife 2020;9:e50060. 10.7554/eLife.50060 32633233 PMC7367686

[34] Public Health Agency of Canada. National Advisory Committee on Immunization (NACI) statement: Seasonal influenza vaccine for 2023–2024. Ottawa, ON: PHAC. [Accessed 2023 Aug 3]. https://www.canada.ca/en/public-health/services/publications/vaccines-immunization/national-advisory-committee-immunization-statement-seasonal-influenza-vaccine-2023-2024.html

